# Fungal Head and Neck Dermatitis: Current Understanding and Management

**DOI:** 10.1007/s12016-024-09000-7

**Published:** 2024-07-20

**Authors:** Albert C. Chong, Francisco José Navarro-Triviño, Malcolm Su, Chang Ook Park

**Affiliations:** 1 Department of Internal Medicine, Mayo Clinic Arizona, 13400 E Shea Blvd., Scottsdale, AZ 85259 USA; 2 Keck School of Medicine, University of Southern California, Los Angeles, CA USA; 3 Department of Contact Eczema and Immunoallergic Diseases, Dermatology Service, Hospital Universitario San Cecilio, Granada, Spain; 4 Department of Internal Medicine, University of Texas Southwestern Medical Center, Dallas, TX USA; 5 Department of Dermatology & Cutaneous Biology Research Institute, Yonsei University College of Medicine, Seoul, South Korea

**Keywords:** Atopic dermatitis, Head and neck dermatitis, Fungi, *Malassezia*, Dupilumab-associated head and neck dermatitis, Th17

## Abstract

Head and neck dermatitis (HND) is a form of atopic dermatitis (AD) that affects the seborrheic areas of the body and causes greater quality of life detriments than other types of AD. HND can be challenging to treat since first-line topical therapies may be ineffective or intolerable for long-term use on areas affected by HND while dupilumab may cause dupilumab-associated HND (DAHND). Current evidence implicates fungi, particularly *Malassezia* spp., in the pathogenesis of HND. Penetration of fungal antigens through the defective AD skin barrier activates the innate and adaptive immune systems to cause cutaneous inflammation via the T helper (Th)17 and/or Th2 axes. *Malassezia* sensitization may distinguish HND from other forms of AD. Multiple double-blind, placebo-controlled trials have shown antifungals to benefit HND, yet the persistence of symptom relief with sustained use remains unclear. Oral antifungals appear more effective than topical antifungals but may be harmful with long-term use. DAHND may also be fungal-mediated given improvement with antifungals and evidence of an overactive immune response against *Malassezia* in these patients. Janus kinase inhibitors are effective for HND, including DAHND, but may cause significant side effects when administered systemically. OX40/OX40L inhibitors and tralokinumab may be promising options for HND on the horizon. Demographic and environmental factors influence the host mycobiome and should be considered in future precision-medicine approaches as microbiome composition and diversity are linked to severity of HND.

## Introduction

AD is a chronic inflammatory and pruritic skin condition with substantial physical, psychosocial, and financial burden for patients and their caregivers [1–6]. HND is a subtype of AD that primarily affects the seborrheic areas of the body including the head, face, neck, and upper trunk [7–9]. Among AD patients, up to 36% of adults and 79% of children may be affected [7, 10, 11]. HND is associated with greater health-related quality of life burden in both adults and children compared to AD of other distributions [12]. Importantly, involvement of the head, neck, and facial regions has a greater impact on quality of life than objective severity score and total body surface area affected [12]. In the context of AD patient input that wearing occlusive clothing benefits their mental health, cosmetic appearance is likely a major contributor to low quality of life in HND given the highly visible distribution of lesions [2, 12, 13]. Two phenotypes may exist including adolescent-onset HND with exclusive head and neck involvement and adult-onset HND, which may accompany diffuse dermatitis [14].

Conventional AD treatments may be suboptimal for HND. Topical corticosteroids are the mainstay of AD management [15] but may cause facial skin atrophy, perioral dermatitis, or topical corticosteroid withdrawal [16–18]. Meanwhile, topical calcineurin and phosphodiesterase-4 inhibitors may improve some HND, but their use is limited by burning and stinging at the application site, which may be particularly bothersome on the face and neck [19, 20]. Since its introduction in 2017, the biologic dupilumab has been life-changing for many AD patients with moderate-to-severe AD refractory to topical medications. However, dupilumab is increasingly documented to exacerbate or even cause HND in a subset of patients, resulting in treatment discontinuation in around 11% of cases [21–25]. Dupilumab-associated HND (DAHND) may affect 29% of children and 6% of adults [26, 27], and no standard treatment for this complication exists [24]. Interestingly, HND is common among AD patients with exacerbations despite biological therapy [28], suggesting that HND represents a refractory and perhaps mechanistically distinct subset of AD.

The microbiome is increasingly accepted as a key factor in AD, and microbiome profiles cluster differently by disease presence and severity [29, 30]. Currently, bacterial contributions (i.e., *Staphylococcus aureus*) are the most explored and recognized [31, 32]. However, growing evidence implicates fungi, especially *Malassezia* spp., in HND [33], and both Th2 and Th17 axis mechanisms have been proposed [34, 35]. Understanding how fungi cause or exacerbate HND may enable more effective and upstream therapies for this disease and guide the development of AD therapeutics without DAHND-type reactions. Our review summarizes the current understanding of fungal contributions to HND and discusses rational medical management of this condition.

### Malassezia

#### *Malassezia* Colonization Patterns

Genetic sequencing has revealed at least 11 fungal genera on human skin including commensals *Malassezia*, *Rhodotorula*, *Debaryomyces*, *Cryptococcus*, and *Candida* [36]. Among cutaneous fungi, *Malassezia* is predominant at 11 core-body and arm sites including the retroauricular crease, nares, and occiput despite differences in species representation between sites [36]. *Malassezia* spp. are lipophilic yeasts dependent on exogenous lipids given their lack of fatty acid synthase [34, 37]. Thus, sebaceous areas of the skin are more favorable for their growth [38–40]. *Malassezia* colonization increases over the first year of life before remaining static [41, 42] until puberty, when progression occurs rapidly due to the increase in androgens and accompanying elevation of sebum production [43]. By adulthood, *Malassezia* spp. represent between 53 and 80% of all fungi depending on the body area and may be found in the highest numbers behind the ear [44]. *Malassezia* colonization increases with age [45], and males show higher cutaneous *Malassezia* abundance [42, 46], likely due to greater sebum production versus females [47, 48].

The environment (i.e., temperature, humidity, air pollution) has an established role in modulating AD, which may include affecting mycobiome composition [31, 33]. Both low and very high temperatures and humidity are known to trigger AD in addition to exposure to indoor heating [31]. In a cohort of Japanese scientists journeying to Antarctica, Sugita et al. found increases in *Malassezia* colonization across body sites and most dramatically on the scalp with predominance of *M. restricta* [49]. Similarly, astronauts traveling to space show an increased ratio of *Malassezia* to all fungi on the skin [50]. Meanwhile, a study comparing Singaporean and Swiss mycobiomes observed that tropical (humid) climates promote *Malassezia* colonization and fungal species diversity [51]. Air pollution including particulate matter and gaseous pollutants is also now a recognized contributor to AD [52–55], and fungal dysbiosis due to these pollutants may represent a possible mechanism for AD worsening with air pollution [56, 57].

Representation of specific *Malassezia* spp. varies with demographic, micro-environmental (i.e., location on body), and macro-environmental (i.e., location on planet) factors. Regarding age and body part, *M. globosa* is more frequently cultured from younger patients and along the scalp and forehead, while *M. sympodialis* is more commonly found in adult patients and along the skin of the back [45]. This may be related to changes in skin lipid and pH profiles with age [48, 58]. Further, the diversity of *Malassezia* may vary across patients of different geographic locations and ethnicities. For instance, Singaporean subjects carry more species of *Malassezia* on average than those living in Switzerland [59, 60]. *M. sympodialis* is commonly isolated from the chest, back, face, and scalp of subjects living in Poland, Sweden, and other temperate climates while *M. globosa* is more encountered in warmer climates [45, 61, 62]. These findings illustrate the variability of *Malassezia* spp. representation among patients, supporting the existence of species-specific niches that may depend on myriad factors including temperature, humidity, ultraviolet exposure, presence of other microorganisms, availability of specific metabolites, and beyond.

*Malassezia* spp. diversity and relative abundance may be relevant to AD. AD lesions show greater diversity of *Malassezia* spp. compared to the skin of healthy individuals [63, 64]. Among AD patients, *M. globosa* and *M. restricta* may be more abundant than *M. sympodialis* [33]. Further, changes in the mycobiome composition may correspond with disease severity [29]. In adult HND patients living within the same geographic region (Japan), the number of *Malassezia* spp. observed is similar across disease severities but the relative abundance of each species varies [58]. HND patients with mild-to-moderate disease show a greater abundance of *M. restricta* versus *M. globosa* colonization in skin lesions [58] similar to between-flare AD skin [65]. In contrast, severe HND lesional skin features equal proportions of the two species [58]. Thus, a rising proportion of *M. globosa* to *M. restricta* could signal a shift towards severe disease in HND.

#### AD Predisposes to Cutaneous *Malassezia* Allergenicity

AD patients exhibit a skin surface pH that is up to 0.9 pH units higher than the mean healthy control skin pH of 5.24 [66, 67]. Alkaline pH not only fosters *Malassezia* growth [18] but also increases *Malassezia* antigen production, for example of the major allergen Mala s 12 by *M. sympodialis* [68]. Meanwhile, AD skin features decreased levels of antimicrobial/antifungal peptides including cathelicidins (i.e., LL37) and β-defensins [69–71], which may bias towards overactivation of Th17 and/or Th2 axis immunity [18]. However, further clarification may be needed since dendritic cells in severe AD have been observed to exhibit enhanced LL37 secretion in response to *M. sympodialis* exposure versus milder AD and healthy controls [72]. Cytokine gene variants (i.e., interleukin(IL)-10 and interferon(IFN)γ) may also increase susceptibility to *Malassezia* infection [73], and these have been associated with AD [31, 74, 75].

According to skin prick tests (SPTs), specific immunoglobulin E (sIgE) tests, or atopy patch tests (APTs), 67% of AD patients may be sensitized to *Malassezia* versus 0% of healthy controls [76]. This is consistent with the increased sensitization to many inhalant, food, and microbial allergens in AD due to the inherent skin barrier defects and increased propensity of atopic patients to mount immune responses to allergens [77, 78]. Among AD patients, *Malassezia* spp. sIgE is found more frequently in males and adults [34, 79], consistent with aforementioned trends of increased *Malassezia* colonization in males and with increased age. Sensitization to *Malassezia* is more common in HND versus other types of AD or healthy controls [80, 81]. In fact, sIgE to *M. furfur* has been reported in up to 100% of HND patients versus 13.6% of AD patients with other lesional distributions [80]. Thus, immunogenicity against *Malassezia* spp. is a typical feature in HND, and HND may be mechanistically distinct from other forms of AD.

Paradoxically, multiple studies have found that cutaneous *Malassezia* is diminished in AD patients versus healthy controls [33, 62, 82]. Moreover, among AD patients, fewer positive *Malassezia* cultures and lower colony-forming unit counts are obtained in lesional versus non-lesional skin [62, 82]. These observations may be explained by the dysregulated immune state of AD that is still microbicidal by nature and may actively suppress *Malassezia* expansion [35, 83]. Indeed, AD patients with positive SPTs or sIgE against *M. sympodialis* are less often positive in *Malassezia* cultures [62], supporting the theory that immune activation against *Malassezia* in AD may explain diminished *Malassezia* numbers in an otherwise favorable environment. Notably, *Malassezia* colonization is two-to-fivefold higher in severe HND skin versus mild-to-moderate HND and healthy skin [58]. We theorize that severe HND may be triggered by periods of *Malassezia* proliferation that potentiate the immune response [35, 72]. As control over *Malassezia* is obtained, the disease may return to a milder severity characterized by smoldering inflammation and lowered *Malassezia* presence.

#### *Malassezia* Pathophysiology

**Fig. 1:**
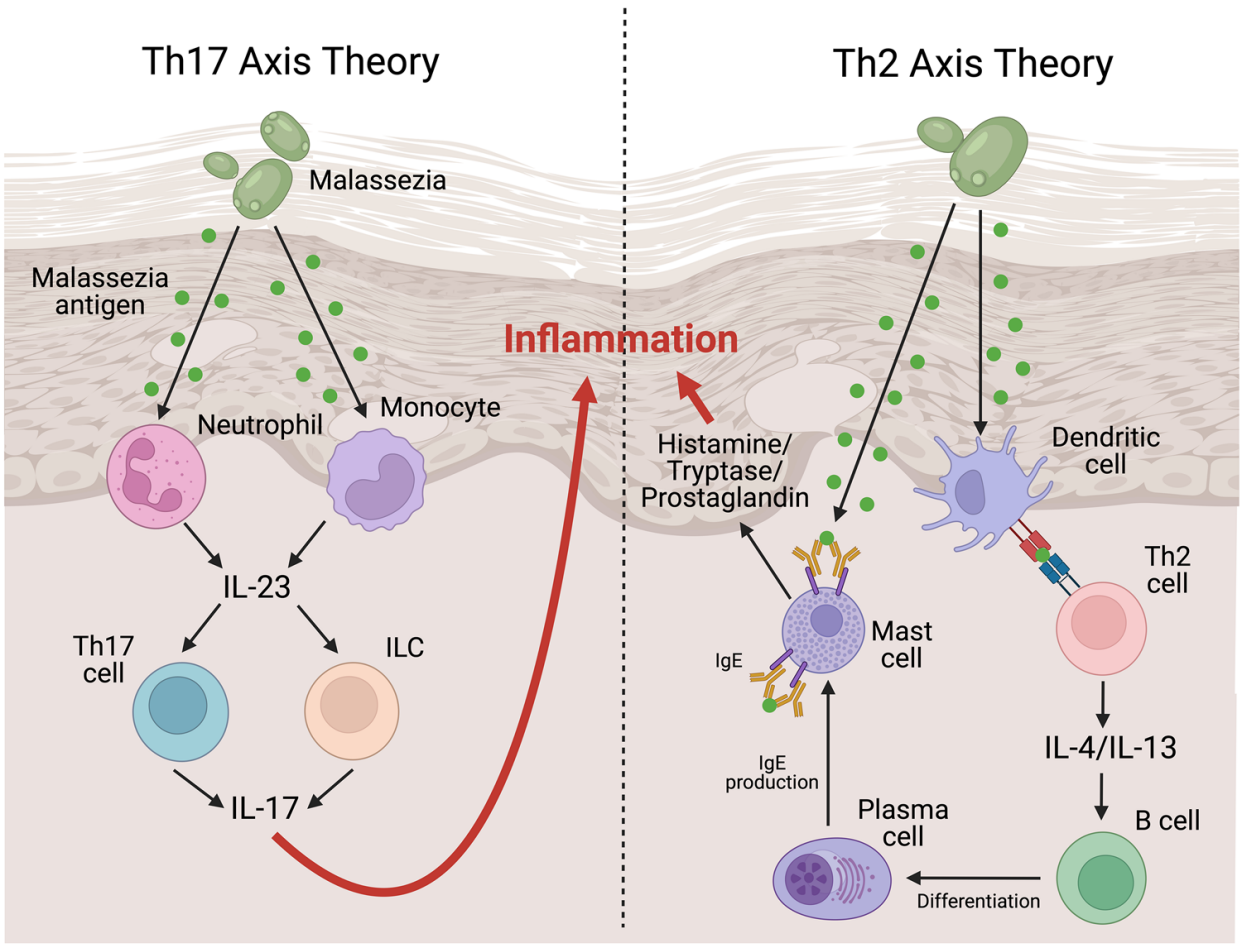
Current mechanistic theories for fungal-driven head and neck dermatitis

*Malassezia* spp. are skin commensals that are normally tolerated by the human immune system [84]. In AD however, the defective skin barrier and hyperactive immune system result in immune activation against the yeast*.* AD is characterized by a compromised skin barrier due to deficient stratum corneum components and decreased keratinocyte differentiation [31]. Compared to non-HND AD and control patients, HND skin is further deficient in ceramides such as esterified omega-hydroxyacyl-sphingosine, which typically maintain skin barrier integrity and have antifungal properties against *Malassezia* [81, 85]. Upon entering the skin, *Malassezia* antigens may trigger cutaneous inflammation through the Th17 and/or Th2 axis (Fig. 1). From a Th17 perspective, *Malassezia* antigen may trigger neutrophil or monocyte release of IL-23, resulting in Th17 or innate lymphoid cell release of IL-17 and a dysregulated inflammatory antifungal response [35]. In support, *Malassezia*-specific Th17 memory T cells are more abundant and reactive in AD patients versus healthy individuals [35]. From a Th2 perspective, dendritic cells may present fungal antigen to Th2 cells, triggering the release of IL4 and IL13, B-cell class switching, maturation to IgE-producing plasma cells, and ultimately an allergic IgE-mediated response [34]. In support, *Malassezia* sIgE levels have been shown to be positively associated with clinical severity of HND [86]. Further, a genotyping study identified four HLA risk alleles and two protective alleles for *Malassezia*-related skin diseases including HND, suggesting that abnormal antigen presentation may have a role [87]. However, Sparber et al. observed that Th2 cytokines including IL-5, IL-13, and TSLP were unchanged or decreased with cutaneous *M. pachydermatis* exposure in mice, which may favor Th17 over Th2 activation as the mechanism of fungal-driven AD.

Various antigens and antigen-specific mechanisms have been proposed for fungal AD. Mala s 11, Mala s 12, and Mala s 13 are potential major allergens for AD patients sensitized to *M. sympodialis* and may result in cross-reactivity with human manganese superoxide dismutase, the glucose-methanol-choline oxidoreductase enzyme superfamily, and human thioredoxin, respectively [34, 88–90]. *M. sympodialis* antigens may also augment AD mast cell release of IL-6 [91], a cytokine known to increase numbers and reactivity of mast cells [92], thus potentiating an allergic response. *M. globosa* MGL_1304 has been identified as a major histamine-releasing antigen in human sweat and may explain why sweating triggers cutaneous symptoms in some AD patients [93]. *M. sympodialis* Mala s 8 and *M. restricta* Mala r 8 are homologous to MGL_1304 and have also been implicated in HND [94]. *M. globosa* glycoprotein Mal g 46b is another potential major antigen in AD patients [95]. Finally, *M. furfur* secretes proteases (i.e., human secretory aspartyl protease MGSAP1) that may contribute to inflammation, increase virulence, and facilitate colonization [96].

### Non-*Malassezia* Fungi

#### Non-*Malassezia* Diversity

Many non-*Malassezia* fungi have been recovered from AD skin, including *Candida*, *Cryptococcus*, *Trichosporon*, *Saccharomyces, Meyerozyma*, *Rhodotorula*, *Debaryomyces*, *Aureobasidium*, *Penicillium*, *and Geotrichum* [29, 97]. In fact, non-*Malassezia* fungi are more diverse in AD skin as compared to the skin of healthy individuals [29, 98]. AD patients have higher rates of *Candida*, *Cryptococcus*, and *Geotrichum candidum* colonization when compared to healthy individuals [29, 97, 99–101]. The proportion of *C. diffluens* and *C. liquefaciens* isolated from AD patients is almost twice that of healthy subjects [102]. Interestingly, severe AD skin is more often colonized with non-*Malassezia* fungi than mild-to-moderate AD skin [30]. In severe disease, the relative abundance of *Malassezia* may be decreased in favor of *Candida*, *Debaryomyces*, *Aureobasidium*, and *Penicillium* when compared against mild-to-moderate AD or healthy controls [30]. Some organisms including *C. albicans*, *C. diffluens*, and *C. liquefaciens* may be unique to AD patients and not present on healthy skin [29].

#### Non-*Malassezia* Pathophysiology

Several non-*Malassezia* species associated with AD have received limited attention for potential roles in disease pathogenesis. One study showed that 35% and 40% of AD patients have sIgE against *Cryptococcus diffluens* and *Cryptococcus liquefaciens*, respectively, suggesting that components of these organisms could play a role in AD or provoke hypersensitivity responses [103]. In patients with AD, the mannan of *C. albicans* may induce elevated IL-2 and IFNγ responses that may contribute to the pathogenesis of the disease [104]. Lastly, though the role of *Trichophyton* in the pathophysiology of AD is not well understood, 79% of AD patients with dermatophyte infections experience an immediate hypersensitivity reaction against *Trichophyton* antigen [105].

## Dupilumab-Associated HND

**Fig. 2:**
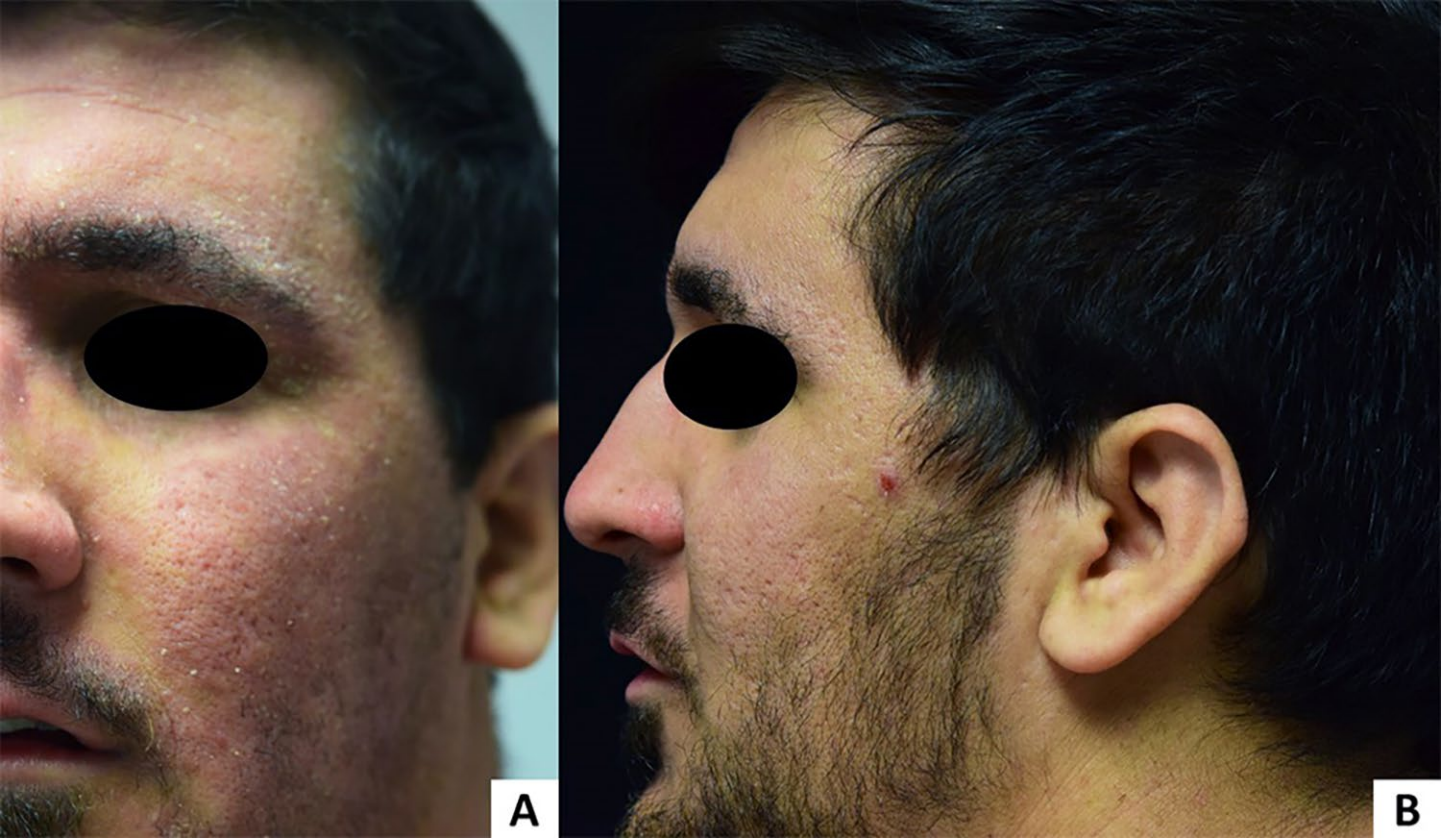
**A** DAHND 3 months after starting dupilumab 300 mg every 2 weeks. **B** Almost complete resolution after treatment with itraconazole 100 mg daily for 21 days. Dupilumab was not discontinued

Dupilumab is an IgG monoclonal antibody that targets the α subunit of IL-4 and IL-13 receptors, blocking the Th2 axis that is central to AD pathogenesis [106]. DAHND is most often reported as new onset or acute worsening of head and facial erythema and pruritus after dupilumab therapy [107] and may occur 10–39 weeks after initiation of dupilumab treatment [108] (Fig. 2). DAHND has also been called new regional dermatoses, dupilumab facial redness, paradoxical head and neck erythema, and persistent facial dermatitis [25]. Biopsy shows ectatic capillaries and perivascular lymphocytic exocytosis without spongiosis [108]. Mast cells, T cells, and histiocytes occur in normal numbers, and plasma cell counts are variable [108]. Meanwhile, only small numbers of eosinophils are present, and B cells, neutrophils, and macrophages are largely absent [108].

A potential explanation for DAHND may be the shift towards Th17/Th22 antifungal immunity in AD patients treated with dupilumab [18, 109–111]. The Th2 cytokine IL-13 is thought to maintain homeostasis between the host and *Malassezia* colonization [18]. Blocking the Th2 pathway with dupilumab may skew immunity towards the highly antifungal Th17/Th22-mediated pathway, generating an immune response against commensal *Malassezia* [18, 110]. In support, a recent cohort study found that *Malassezia* colonization on the forehead decreased following dupilumab administration despite continued predominance of *M. restricta* and *M. globosa* over the cutaneous mycobiome [112]. Meanwhile, in a punch biopsy study of human DAHND skin where DAHND was defined as de novo HND following dupilumab commencement, CCR7 ligand *CCL21* was upregulated in DAHND smooth muscle cells and contributed to Th17 chemotaxis [109, 113]. Th17 overactivation against *Malassezia* is further supported by increased *Malassezia*-specific Th17 memory T cells in the blood of AD patients [35]. Meanwhile, DAHND skin biopsy also revealed increased Th22 markers in oligoclonal expanded T cells and enhanced keratinocyte activation with IL-22 receptor upregulation [109]. Notably, a prospective study by Kozera et al. showed that patients who develop DAHND have significantly greater levels of serum *Malassezia* sIgE compared to those who do not (median 31.95 kU/Lversus 2.27 kU/L; *P* = 0.005) despite no differences in serum total IgE between groups at baseline (4570 kU/L versus 4478 kU/L; *P* = 0.95) [24]. This study defined DAHND as de novo or worsening HND after starting dupilumab (worsening was defined as 50% worsening in head and neck Eczema Area and Severity Index (EASI) score). Further research is necessary to confirm if *Malassezia* sIgE plays a direct role in DAHND or if it may function as a biomarker of disease severity.

From a treatment perspective, Kozera et. al showed that DAHND may temporarily improve with oral itraconazole therapy and that *Malassezia* sIgE levels mirror clinical improvement, supporting hyperactive immunity against *Malassezia* spp. as a feature of DAHND (Fig. 2). Interestingly, the anti-IL13 antibody tralokinumab improves AD in some patients who developed HND with dupilumab [114] and may be safe and effective for short-term use in HND [115]. Thus, IL-13 blockade alone may not be sufficient to trigger Th17/Th22 overactivation, and IL-4 or IL-4 receptor α blockade may be required as with dupilumab. Other proposed mechanisms for DAHND include underlying contact dermatitis, rosacea via *Dermodex* proliferation, and psoriasiform dermatitis, although these have not been well-studied [116].

## Diagnosis

Clinically, *Malassezia* hypersensitivity may be suspected in patients with AD and head and neck lesions [117]. AD exacerbations during adolescence or young adulthood, severe lesions recalcitrant to conventional therapy, and comorbid atopic disease may be supportive features [117]. Dupilumab initiation up to 9 months prior to symptom onset may be considered suggestive of DAHND [108]. Sensitization tests may be useful to determine the etiology of HND, although *Malassezia* sensitization accompanies most HND [18, 86]. Currently, SPT, sIgE testing, and APT for *Malassezia* spp. [76] are not established components of the AD workup, but positivity may lend support to a diagnosis of fungal HND. Polymerase chain reaction (PCR) is another promising tool for diagnosis of fungal HND and offers rapid (around 72–96 h if sequencing is performed for species identification) and specific results. In relation to *Malassezia* spp., the main molecular target for PCR is internal transcribed spacer 1 (ITS1), which allows for sequencing to identify specific species. Skin scales obtained through scraping or tape-stripping the affected area are deposited in a polypropylene container with an airtight lid. These samples may be refrigerated for a maximum of 2 days, while freezing allows for extended conservation. Another benefit of PCR is that it allows detection of single nucleotide polymorphisms and thus analysis of possible resistance to azoles and other antifungals. Finally, microscopic visualization of *Malassezia* via tape or direct impression smear [118] may also support diagnosis of fungal HND, yet the utility of this method is questionable without species differentiation since *Malassezia* are common skin commensals. Should non-*Malassezia* fungi prove to be relevant contributors to HND, similar methods may be leveraged for identifying hypersensitivity and/or presence of non-*Malassezia* fungi.

## Management

While topical first-line therapies for AD (i.e., corticosteroids, PDE4 inhibitors, calcineurin inhibitors) may be intolerable or ineffective in HND, promising options include antifungal agents and more recently, JAK inhibitors, OX40/OX40L inhibitors, and anti-IL-13 therapy. Currently, European guidelines for AD recommend topical (ketoconazole or ciclopirox olamine) or systemic (itraconazole or fluconazole) antifungal therapy for AD patients with HND, especially those with clear IgE sensitization to *Malassezia* spp. [119]. Some clinicians may add tacrolimus, as there is evidence of synergism with antifungals against *Malassezia* [120]. Pyrithione zinc, butenafine, and lactic acid topicals can be considered since they may decrease *Malassezia* burden [60, 121, 122], although their efficacy for HND is not well-established.

Oral antifungal agents have shown promising efficacy in the treatment of AD patients with *Malassezia* allergy. These agents have been associated with improved SCORing Atopic Dermatitis (SCORAD) scores, decreased topical steroid use, decreased *Malassezia* sIgE, and decreased total serum IgE [117, 123, 124]. A double-blind, placebo-controlled trial (DBPCT) showed that 200 mg oral ketoconazole for 30 days improves HND dermatitis with *M. furfur* sensitization [124]. Another DBPCT showed that 200 or 400 mg itraconazole monotherapy for 1 week improves HND severity for up to 6 weeks [123]. DAHND may also benefit from oral itraconazole therapy as observed over 4 weeks [125]. Currently, the persistence of HND relief with sustained oral antifungals remains unknown, and side effects must be considered as long-term systemic azole use may cause hepatotoxicity and hormone-related irregularities [126]. European guidelines recommend imidazole derivatives (i.e., fluconazole or itraconazole) over ketoconazole for systemic treatment given their superior benefit to side-effect ratios [119].

While topical antifungals may have lower penetration into *Malassezia* reservoirs (i.e., the hair follicle) and less compliance compared to systemic options [117], some evidence supports their use in HND. A DBPCT of HND patients showed significant improvement in investigator global assessment (IGA) score after 1 month of ciclopirox olamine cream applied twice daily (− 1.2 versus − 0.7 for placebo; *P* = 0.049) and a similar trend in EASI score (− 128.2 versus − 98.8 for placebo; *P* > 0.05) [127]. Efficacy was supported by rebound HND symptoms upon discontinuation of ciclopirox olamine resulting in worsened IGA (0.4 versus − 0.2 for placebo; *P* = 0.025) and EASI (37.7 versus 0.6 for placebo; *P* > 0.05) scores at 2 weeks [127]. Notably, *M. furfur* isolates may be more drug tolerant to azoles and ciclopirox olamine than other *Malassezia* spp. [61]. Meanwhile, topical ketoconazole applied twice daily was observed to improve facial rashes refractory to dupilumab in AD patients [116]. Another DBPCT found no difference between topical miconazole-hydrocortisone cream and ketoconazole shampoo versus hydrocortisone alone for head and neck AD [128]. Since both groups showed improvement in this study, the effect of topical antifungals alone could not be ascertained. Notably, topical ketoconazole twice daily has also been observed to improve DAHND [18].

Pooled phase 3 clinical trials have shown that oral JAK inhibitors such as baricitinib significantly improve HND severity in moderate-to-severe AD [17]. Baricitinib inhibits JAK1/JAK2 signal transduction in AD to decrease downstream release of cytokines including IL-5, which is thought to improve AD by inhibiting eosinophil activation and migration to the skin [129, 130]. These medications may also improve DAHND, as upadacitinib monotherapy following oral itraconazole produces sustained improvement for DAHND [125]. Importantly, oral JAK inhibitors may cause significant side effects including opportunistic infections (i.e., tuberculosis), reactivation of latent infections (i.e., herpes zoster), and increased risk for non-melanoma skin cancer [131]. Consequently, they should be reserved for cases when other methods fail or are contraindicated. For HND in mild-to-moderate AD, phase 2 and 3 trials suggest that topical JAK inhibitors such as ruxolitinib significantly improve severity, itch, and quality of life [132]. Topical JAK inhibitors carry lower risk of infectious complications compared to systemic therapy and application site reactions are rare, even in HND regions [132, 133].

**Fig. 3:**
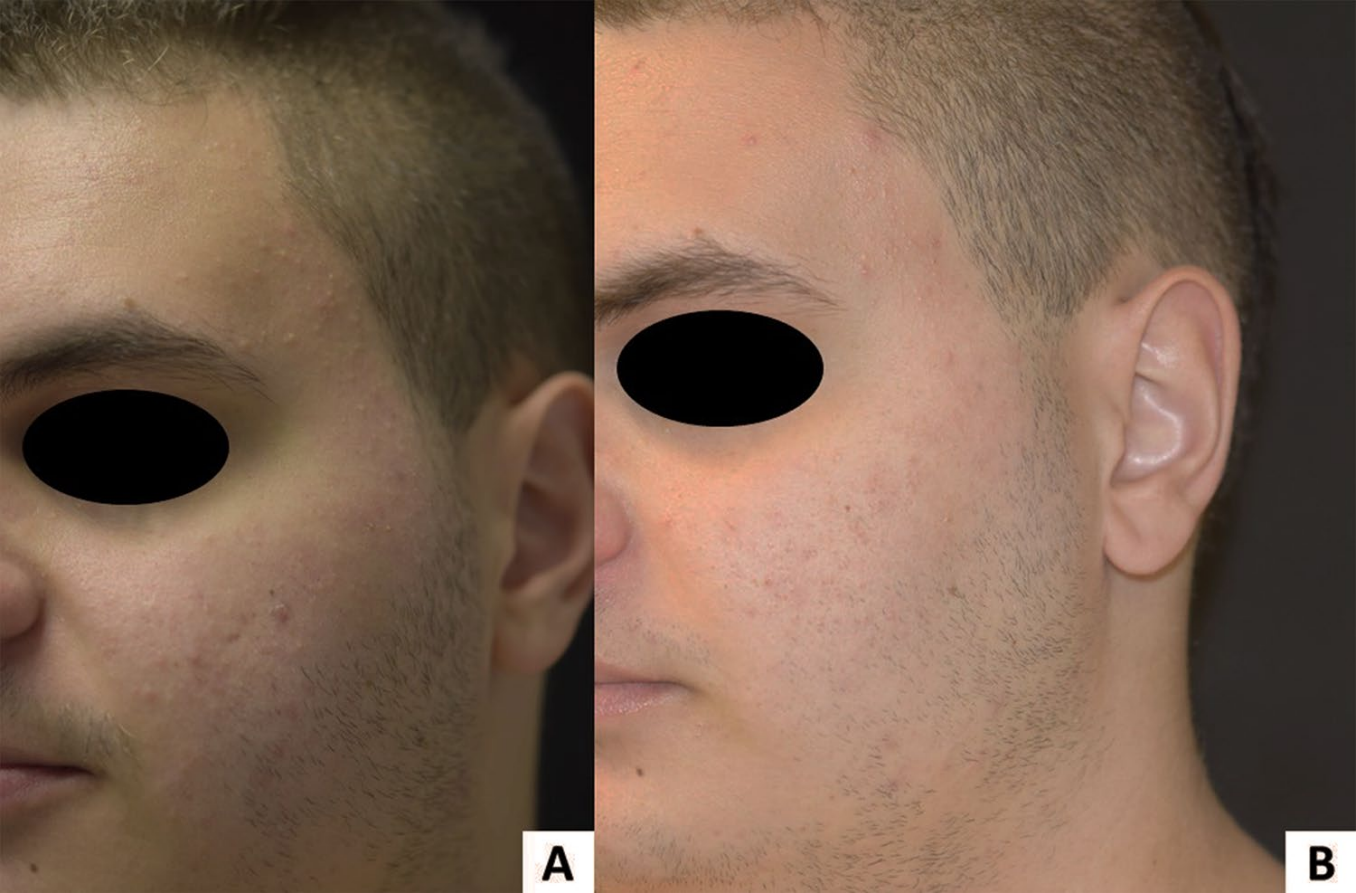
**A** Facial acneiform eruption 6 weeks after starting abrocitinib 200 mg daily. **B** Almost complete clearance of the acneiform eruption after application of ketoconazole 2% cream twice daily for 21 days. Abrocitinib was not discontinued

**Fig. 4:**
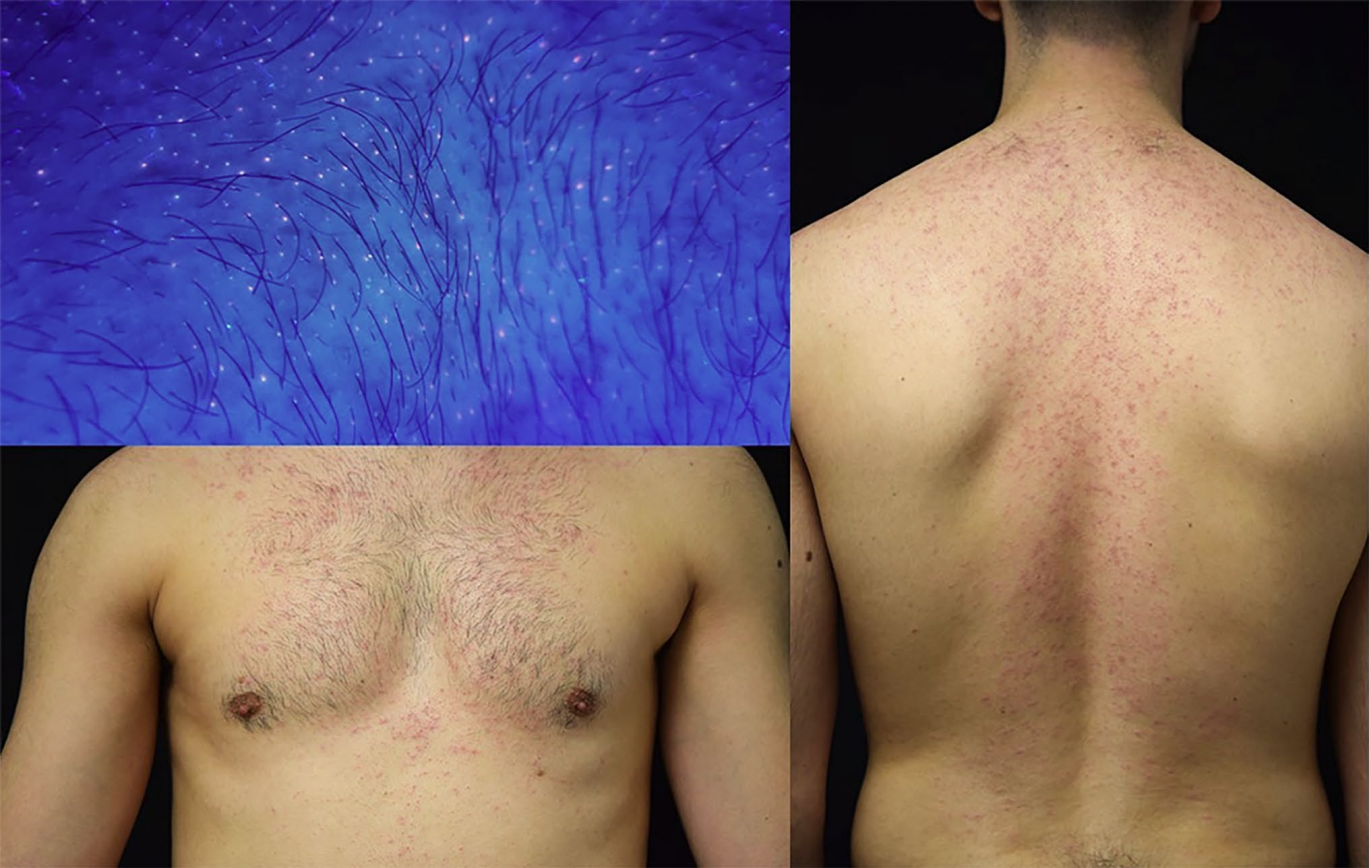
Truncal acneiform eruption 8 weeks after starting upadacitinib 30 mg daily. Yellow-orange combined with red fluorescence on Wood’s light examination compatible with *Malassezia* spp. and *Cutibacterium acnes*

Curiously, acneiform eruption has been associated with JAK inhibitor therapy [134, 135], representing another potentially fungal-mediated rash secondary to AD treatment. This “JAKne” occurs in up to 14% of patients on systemic JAK inhibitors in a dose-dependent manner with upadacitinib and abrocitinib showing a higher incidence than baricitinib [135, 136]. JAKne appears in the first months after starting treatment and presents as follicular and non-follicular erythematous papules most commonly on the face (Fig. 3) but also on the trunk (Fig. 4) [137]. Wood’s light shows a predominant yellow-orange fluorescence (compatible with *Malassezia* spp.), combined with red fluorescence of variable degree (compatible with *Cutibacterium acnes*). This examination is an important clinical finding of dysbiosis associated with JAK inhibitors, which can be confirmed by microbiological study. The dramatic improvement with oral antifungals (i.e., itraconazole or fluconazole) is another clinical finding that supports the suspicion of dysbiosis due to *Malassezia* spp. as a microorganism responsible for the acneiform eruption caused by these drugs (Figs. 3 and 4).

OX40/40L inhibitors and anti-IL-13 therapy may be other options for HND on the horizon. Since the pathogenesis of HND involves T cell subsets expressing OX40, therapies targeting OX40/40L (i.e., rocatinlimab and amlitelimab) are logical options that act via modulation of effector T cell survival and expansion [138]. Indeed, HND patients with moderate-severe disease treated with rocatinlimab have shown improvement in severity compared to placebo in phase 2 trials [139] and rocatinlimab has been generally well-tolerated [138]. Finally, the anti-IL-13 monoclonal antibody tralokinumab appears safe and effective for HND for 16 weeks and may even treat cases of DAHND as mentioned above. More extensive clinical trials are needed to evaluate OX40/40L inhibitors and tralokinumab for HND.

## Conclusion

HND presents unique challenges and may deviate from other forms of AD regarding etiology and optimal management strategies. The involvement of *Malassezia* spp. in HND is highly likely given enhanced sensitization to *Malassezia* among HND patients, demonstrated AD exacerbation upon *Malassezia* exposure in animal studies, and clinical improvement of HND with antifungals in human trials. Current understanding and management of fungal HND and DAHND is incomplete. For example, there remains controversy regarding HND pathophysiology (i.e., Th17 versus Th2 theories), and the contribution of non-*Malassezia* fungi is largely unknown. Alternative mechanisms for DAHND are also poorly studied. From a management perspective, the number of studies for treatment of HND and especially DAHND remains quite limited. Further clinical trials are necessary to confirm the safety and efficacy of existing therapies (i.e., antifungals, small molecules, and biologics) for HND, to determine optimal dosage and timing, and to evaluate combination therapies (i.e., antifungals plus immunosuppression). JAK inhibitors, OX40/40L inhibitors, and anti-IL-13 therapy are promising advanced therapies for HND. Finally, precision-medicine approaches for HND should consider demographic and environmental factors as they may influence the mycobiome composition and in turn affect disease severity. Thus, while the mechanisms and therapies for fungal HND require continued study and refinement, the identification of fungi as a major contributor to HND will perhaps enable a renaissance of better understanding and rational management for this disease.

## Data Availability

Not applicable.
